# Exploring the biopsychosocial impact of hypermobility spectrum disorders and Ehlers-Danlos syndrome in an adult population: a protocol for a scoping review

**DOI:** 10.1186/s13643-024-02452-0

**Published:** 2024-01-12

**Authors:** Natalie L. Clark, Melissa Johnson, Amar Rangan, Katherine Swainston, Lucksy Kottam

**Affiliations:** 1 South Tees Hospitals NHS Foundation Trust, Middlesbrough, UK; 2 The Mary Kinross Trust & RCS Chair, Department of Health Sciences & Hull York Medical School, University of York, York, UK; 3 Faculty of Medical Sciences, Newcastle University, Newcastle Upon Tyne, UK

**Keywords:** Joint hypermobility, Biopsychosocial, Psychology, Scoping review

## Abstract

**Background:**

Conditions such as hypermobility spectrum disorders (HSD) and Ehlers-Danlos syndrome (EDS) are most often diagnosed when an individual has joint flexibility beyond the normal physiological limits. Additional characteristics and symptoms include pain and fatigue with individuals also being more likely to report feelings of anxiety and depression. Due to the varied presentation of these conditions, there is a lack of understanding amongst the various healthcare professionals (HCPs) individuals present to, leading to delayed diagnoses and negative experiences for the individuals themselves. This scoping review therefore aims to map the known biopsychosocial impact of adults with HSD and EDS.

**Methods:**

The scoping review will follow the six-step framework as outlined by Arskey and O’Malley and the Preferred Reporting Items for Systematic Reviews and Meta-Analyses for Scoping Reviews (PRISMA-ScR) checklist. The search will be conducted using the following databases: AMED, CINAHL, Cochrane Library, Embase, MEDLINE, PsycINFO, PubMed PEDro. Full-text published articles in the English language (excluding literature and systematic reviews) with adult samples (over the age of 18 years) and a diagnosis of a HSD or EDS, published between 2012 and 2022, will be included in the review.

**Discussion:**

This review will aim to explore the existing literature for the reported biopsychosocial impact of adults with a HSD or EDS. It will also aim to further acknowledge the gaps in understanding of the condition, how the condition and the impact of the condition is being measured and what HCPs are involved in supporting such individuals. These gaps will be used to inform a future systematic review. It is the overall goal to increase the knowledge of HCPs and the quality of life of adults living with a joint hypermobility condition.

## Background

Joint hypermobility affects approximately 30% of the population [1] and is characterised by joints that are able to move actively and/or passively beyond the normal physiological limits [2]. Conditions associated with joint hypermobility include hypermobility spectrum disorders (HSD) and Ehlers-Danlos syndrome (EDS) [3, 4], with prevalence varying from 0.2% in Wales [5] to 3% in the UK [6]. Alongside flexibility of the joints, another common accompanying physical complaint from those with hypermobility is joint pain [4]. Individuals with hypermobility can experience a broad range of symptoms, meaning presentations often vary from person to person. It is because the symptoms are so varied that patients can be seen by and referred to a number of different healthcare professionals (HCPs); examples include orthopaedic clinicians [7], rheumatologists [8] and physiotherapists [8].

These connective tissue disorders are lifelong conditions that affect individuals across physical, psychological and social levels with a number of daily consequences [9]. A focus group by Terry et al. [8] provided invaluable insights into the lived experiences of individuals with joint hypermobility syndrome (JHS), highlighting the negative biopsychosocial impact of the condition. The group reported unpredictable and debilitating symptoms of fatigue and pain. As a result of these symptoms, participants would subsequently experience pain catastrophising, increased levels of anxiety and activity restrictions. Due to a lack of understanding of JHS amongst HCPs, the sample experienced delays to diagnosis which cause them to feel fraudulent and stigmatised. This finding has been supported and further explained in that pain and fatigue are non-observable symptoms [10]. Once diagnosed, however, the participants in the focus group described their symptoms feeling validated and were ultimately psychologically relieved.

Research has further evidenced that individuals with HSD and EDS are much more likely to experience psychosocial implications such as anxiety, depression and a lower health-related quality of life [11, 12]. Furthermore, these patients have demonstrated a significantly lower level of participation in societal activities when compared to a healthy group of individuals [13]. This can be attributed to the aforementioned debilitating symptoms such as chronic pain in patients with HSD/EDS. In addition, an individual’s pain experience tends to be exacerbated by comorbid psychological conditions [9]. Despite the literature citing the significance of these psychosocial factors [10], little effort has been made to develop an in-depth understanding of these factors and how to effectively manage them, with the focus primarily being on a physical level. Developing the understanding of HSD and EDS on a broader, biopsychosocial level is essential in order to increase awareness amongst HCPs, allowing them to provide better quality of care and the necessary psychological support [10]. This has the potential to improve the overall quality of life for the individual living with this long-term condition [9].

The primary objective of this scoping review is to therefore systematically scope the known biological, psychological and social (biopsychosocial) impact of HSD and EDS in an adult population. Increasing the understanding of the impact will benefit HCPs and the individual’s themselves and allow for the development of appropriate interventions to assist management and improve information provision [10, 12].

A preliminary search for existing scoping and systematic reviews on hypermobility within the last 10 years in the English language was conducted using a single electronic database, MEDLINE. The search terms used were “joint hypermobility” OR “Ehlers-Danlos syndrome” AND “scoping review” OR “systematic review”. The search generated 401 articles, following screening of the titles, and 34 articles were relevant to the above criteria. Just one scoping review was retrieved relating to urogenital and pelvic complications in EDS and associated HSD [14]. The remaining 33 articles were systematic reviews on various topics relating to joint hypermobility and EDS. Therefore, there is limited evidence, if not no evidence, for previous scoping reviews on joint hypermobility as a whole, including a lack of reviews that examine the biopsychosocial impact exclusively.

## Methods

### Methodological framework

The scoping review will adhere to the framework as developed by Arskey and O’Malley [15]: (1) Identifying the research question, (2) identifying relevant studies, (3) study selection, (4) charting the data and (5) collating, summarising and reporting the results. The sixth step, consulting with stakeholders to inform or validate findings, is optional and is not planned for this scoping review. Extraction and selection of the results will comply with the checklist as outlined by the Preferred Reporting Items for Systematic Reviews and Meta-Analyses for Scoping Reviews (PRISMA-ScR) [16].

#### Step 1: Identifying the research question

The primary research question for the scoping review was underpinned by the PCC (population, concept, context) framework [17]: “What evidence exists on the biopsychosocial impact of EDS and HSD in the adult population?”.

To support the primary research question, the following four objectives of the scoping review were devised:

1. To map the known biopsychosocial impact of HSD and EDS in the adult population within the literature
2. To identify the types of studies (e.g. qualitative) used to identify the biopsychosocial factors
3. To identify how these biopsychosocial factors are measured and managed
4. To describe HCPs involvement

#### Step 2: Identifying relevant studies (search strategy)

Relevant studies will be identified using the following electronic databases: AMED, CINAHL, Cochrane Library, Embase, MEDLINE, PsycINFO, PubMed and PEDro. Additional searches for clinical trials and study protocols will be conducted in ClinicalTrials.gov, EU Clinical Trials Register and ISRCTN. A secondary search of the reference lists of included studies will also be hand searched for any relevant additional studies. The search terms used for the search strategy will relate to two overarching keywords, “hypermobility” (including benign joint hypermobility, Ehlers-Danlos syndrome, hypermobile, hypermobility spectrum disorder, joint hypermobility, joint hypermobility syndrome, generalised joint hypermobility) and “biopsychosocial” (including lived experience, psychological, psychosocial, psychology, social, symptoms, quality of life), combined with Boolean terms such as “AND” and “OR”.

An experienced academic librarian will assist with a pilot search strategy. The pilot search will be conducted to determine suitability of the two keywords and associated terms and electronic databases. Appropriate refinements to the databases, terms and overall search strategy will be made where necessary. The pilot search will be conducted between two authors (N. C. and M. J.) to aid refinement to the search strategy.

#### Step 3: Study selection

##### Eligibility criteria

The scoping review will be guided by the population, concept and context (PCC) framework recommended by Joanna Briggs Institute (JBI) [17]. See Table 1 for additional information.

**Table 1:** PCC framework and study designs

Criteria	Characteristics
Population	• Adults (aged 18 years and older) • Clinical diagnosis of a joint hypermobility condition (e.g. EDS, HSD) as confirmed by a validated tool (e.g. Brighton diagnostic criteria or Beighton score) administered by an appropriate HCP
Concept	Studies that aim to explore the biological, psychological, and/or social (biopsychosocial) impact of adults with a joint hypermobility condition
Context	Samples across all sectors (primary care, secondary care, inpatient, outpatient, rehabilitation, etc.)
Types of evidence	• Study designs including qualitative, cross-sectional, case studies, etc. (excluding literature and systematic reviews only) • Studies published in all countries • Studies published between 2012 and 2022 • Studies available in the English language

The population will include adults (18 years old or over) with a clinical diagnosis of a joint hypermobility condition such as EDS (inclusive of subtypes) or HSD (e.g. JHS, benign joint hypermobility syndrome (BJHS)). The diagnosis must be confirmed by a validated tool (e.g. Brighton diagnostic criteria or Beighton score) administered by an appropriate HCP (i.e. self-reported diagnosis will not be included). The core concept refers to the biopsychosocial impact, broadly looking at the individual biological, psychological and social implications of the conditions and how they affect one another. Context will include all settings (e.g. primary care, secondary care) for the review.

##### Inclusion criteria

To be eligible for the review, articles must meet the following criteria:

- Adult participants (18 years and older) with a clinical diagnosis of a joint hypermobility condition (e.g. HSD, EDS)
- Study designs (e.g. cross-sectional, qualitative, case studies) investigating a biological, psychological and/or social impact of the condition
- Recent literature published between 2012 and 2022
- Articles reported in the English language

##### Exclusion criteria

Articles meeting the following criteria will be excluded:

- Inaccessible full-text articles
- Full-text articles unavailable in the English language
- Systematic or literature reviews

##### Selection process

The articles retrieved using the outlined search strategy will be exported and screened to Microsoft Excel according to the eligibility criteria. Two authors (N. C. and M. J.) will screen the titles and abstracts of studies, and any duplicates will be removed. Full texts will be retrieved for all eligible articles to proceed with further screening against the eligibility criteria. Reasons for exclusions throughout the screening process will be recorded. Any disagreements or clarifications of whether an article is eligible will be resolved by a third reviewer (K. S.). Where full-text articles cannot be accessed, they will also be excluded. The implemented search strategy and the study inclusion process will be reported in full in the scoping review as mapped using the PRISMA flow diagram [18]. Data extraction will be commenced by two authors (N. C. and M. J.); see “Step 4: Charting the data”.

#### Step 4: Charting the data

The data will be extracted according to the draft in Tables 2, 3 and 4; further details of the headings will be described in “Step 5: Collating, summarising and reporting the results”. During the pilot phase, authors N. C. and M. J. will attempt data extraction according to the drafts proposed in the protocol, where necessary, revisions to the data extraction tables will be made. The two authors will meet regularly to discuss the data extraction process. Authors of included studies will be contacted to request missing or clarification of data as and where required (e.g. to confirm age of the sample).

**Table 2:** Study characteristics

Heading	Data extraction
Study details	Author, year, country
Study design	Qualitative, case study, observational, cross-sectional, case control, etc
Participant characteristics	Sample size (*n*), percentage of female participants, mean age and standard deviation, diagnosis of a joint hypermobility condition (e.g. HSD, EDS), recruitment dates

**Table 3:** The biopsychosocial impact of adults with a joint hypermobility condition

Heading	Data extraction
Study details	Author, year
Physical (biological)	Symptoms and conditions, categorised into specialties (e.g. gastroenterology, cardiology)
Psychological	Symptoms and conditions, categorised into specialties (e.g. mood disorders, anxiety disorders)
Social	Impact, categorised into themes (e.g. social networks, daily activities)
Findings	Significant study findings

**Table 4:** Measurements used, treatment/management options and HCP involvement

Heading	Data extraction
Study details	Author, year
Measurements	Reported outcome measures used to assess the biopsychosocial impact (e.g. anxiety and depression — Hospital Anxiety and Depression Scale)
Management	Reported treatment and management options
HCP involvement	Reported HCP involvement (e.g. gastroenterologists, cardiologists)

#### Step 5: Collating, summarising and reporting the results

The results of the scoping review will be presented as a narrative summary with the aforementioned tables presented within the text. The narrative summary and tables will describe and discuss the relevance of the scoping review findings to the primary research question and objectives as previously outlined within the charting the data step. Any gaps identified within the literature will be sufficiently acknowledged, and recommendations for future directions will be sufficiently summarised.

The primary research question (what evidence exists on the biopsychosocial impact of adults with HSD or EDS?) and first objective (to map the known biopsychosocial impact of HSD and EDS in the adult population) will be presented as a narrative summary and in further detail within Table 3. The data will be extracted from studies according to the following: (1) author and year; (2) physical, (3) psychological and (4) social manifestations; and (5) findings. The narrative summary will focus on discussing the prevalence, significance and associations of physical and psychological symptoms, conditions and social impact according to specific categories (e.g. gastroenterology, mood disorders, social networks).

The second objective (to identify the types of studies used to identify the biopsychosocial factors) will be described narratively within text and displayed in Table 2 as study characteristics. The table will include the following: (1) author, year and country, (2) study design and (3) participant characteristics (sample size, percentage of female participants, mean age and standard deviation, diagnosis of a joint hypermobility condition, recruitment dates). This will provide a summary of the current study designs and samples used to demonstrate the biopsychosocial impact.

The third objective is to identify how these biopsychosocial factors are measured and managed, and the fourth objective is to describe HCP involvement. These final objectives will be described throughout the narrative summary and collated within Table 4. The table will include the following: (1) author and year, (2) HCP involvement, (3) measurements and (4) management or treatment. This will provide insight into the varying HCPs involved in the care of individuals with a joint hypermobility condition and the current management and treatment options offered for the condition and its associated symptoms. It will also indicate how the biopsychosocial impact of the condition is measured and whether there is scope to develop a more appropriate outcome measure.

## Discussion

The primary research question and objective of the scoping review are to explore and understand the biological, psychological and social (biopsychosocial) factors and impact of joint hypermobility within adults as reported within the literature. Due to the inclusive eligibility criteria and wide range of terms proposed to use for the search strategy, the review will seek to additionally record measurements used, management of the condition and the HCPs involved as reported by the included studies. This extensive data collection and recording will assist in increasing the understanding of the condition beyond the primary research question, aiming to meet the additional outlined objectives of the scoping review. This will be the first scoping review for the biopsychosocial impact of joint hypermobility conditions. The findings of this review will be used to increase the understanding of the condition for both HCPs and individuals, develop a holistic assessment and inform appropriate pathways to effectively manage the condition.

### Limitations

The scoping review aims to cover a broad range of areas within hypermobility and a wide range of study designs. Depending on the journal articles available, this may be difficult to collate and summarise. The pilot phase should ensure the latter is mitigated to an extent.

## Data Availability

Not applicable.

## Abbreviations

BJHS: Benign joint hypermobility syndrome
EDS: Ehlers-Danlos syndrome
HCPs: Healthcare professionals
HSD: Hypermobility spectrum disorders
JHS: Joint hypermobility syndrome
PRIMSA ScR: Preferred Reporting Items for Systematic Reviews and Meta-Analyses for Scoping Reviews
